# Transient Andes orthohantavirus RNA detection in an asymptomatic healthcare worker following indirect exposure during hospital care of an imported case, the Netherlands, 2026

**DOI:** 10.2807/1560-7917.ES.2026.31.31.2600580

**Published:** 2026-08-06

**Authors:** Alma Tostmann, Annelies Brilman, Loek Smits, Bram Huizer, Chantal Reusken, Margreet te Wierik, Helma Ruijs, Corine Geurts van Kessel, Marloes Mattijssen, Manon Tingen-Wieland, Olga Coenen-Bouwman, Matthew McCall, Esmée Ruizendaal, Ingrid van Schalen, Karin Ellen Veldkamp, Rosa van Mansfeld, Annemiek van der Eijk, Heiman FL Wertheim, Chantal P Rovers

**Affiliations:** 1 Department of Medical Microbiology, Radboud University Medical Center (Radboudumc), Nijmegen, The Netherlands; 2 Center for Infectious Disease Control, National Institute for Public Health and the Environment, Bilthoven, the Netherlands; 3 Department of Internal Medicine, Radboudumc, Nijmegen, The Netherlands; 4 Radboudumc Center for Inflammation, Infection and Immunity, Radboudumc, Nijmegen, The Netherlands; 5 Virology Section, Division Infectious Diseases & Immunology, Department of Biomolecular Health Sciences, Faculty of Veterinary Medicine, Utrecht University, Utrecht, The Netherlands; 6 Department of Viroscience, Erasmus University Medical Center (ErasmusMC), Rotterdam, The Netherlands; 7 Municipal Health Service Gelderland-Zuid, Nijmegen, The Netherlands; 8 Department of Medical Microbiology & Infection Control, LUCID, Leiden University Medical Centre, Leiden, The Netherlands; 9 Department of Medical Microbiology, Amsterdam University Medical Centre, Amsterdam, The Netherlands; * These authors contributed equally to this work and share last authorship

**Keywords:** occupational health, infection prevention and control, virus transmission, Andes hantavirus

## Abstract

Following an outbreak of Andes hantavirus (ANDV) infections on a cruise ship in May 2026, a healthcare worker not directly involved in caring for an imported ANDV case in the Netherlands had a single low-positive ANDV PCR result. The healthcare worker remained asymptomatic, showed no seroconversion, and all subsequent samples tested negative. We describe a retrospective investigation, including detailed reconstruction of potential transmission routes. Our finding may indicate that asymptomatic ANDV infections with low-level viraemia can occur after indirect exposure.

On 2 May 2026, a cluster of severe respiratory illness of unknown origin on the Dutch cruise ship m/v *Hondius* was reported to the World Health Organization (WHO) and the European Centre for Disease Prevention and Control (ECDC) [1]. Andes virus (ANDV) infection was confirmed as the cause of the outbreak that resulted in 13 cases (12 confirmed and one probable) with three deaths [2,3].

On 7 May, a patient with ANDV infection was admitted to Radboud university medical centre, the Netherlands. The patient stayed in a negative air pressure room with anteroom on the infectious diseases (ID) ward until transfer to the intensive care unit (ICU) the same day. For this high consequence infectious disease (HCID), personal protective equipment (PPE) included a filtering face piece FFP-2 mask (similar to N95 or KN95 standards), long-sleeved gown, gloves and face shield.

## Possible exposure incident for healthcare staff

On 9 May, it was identified that advice to use an FFP2 mask when transporting and placing the patient’s urinal in the bedpan washer–disinfector was accidentally not included in infection prevention and control (IPC) protocols. The patient’s urine had tested positive for ANDV RNA on 7 May (quantification cycle (Cq) value = 34). Seven nurses (one from the ID ward and six from the ICU) were consequently considered potentially exposed to aerosols from the urine of the patient. After multidisciplinary national and international consultation, and in a context where detailed guidance on risk assessment was lacking, these exposures were classified as 'high risk' on 11 May. According to the high-risk exposure protocol used in this outbreak, these seven nurses were then placed in quarantine for 42 days after last possible exposure. Monitoring included daily symptom assessment and temperature measurement, weekly PCR testing on EDTA whole blood and plasma, weekly serology, and additional PCR testing of blood, urine and nasopharyngeal samples if symptoms developed [4].

## Single time point low-level Andes virus RNA detection in whole blood

When the Dutch national ANDV guidance became available, the nurses’ exposure risk was reclassified as 'low risk' after multidisciplinary consultation on 27 May, as there was no evidence of exposure to urine aerosols or droplets [5]. However, implementation was postponed when on that same day, the PCR result of a sample taken on 19 May (12 days after potential exposure) from one of the nurses (a healthy man in his 20s; HCWx) was confirmed positive for ANDV RNA. Laboratory contamination was unlikely, as this low-level ANDV RNA load was detected in two samples that were taken independently, processed separately, and that were tested in two different laboratories (at the Dutch Institute for Public Health and the Environment and at Erasmus University Medical Center) with two different PCR tests (a 'pan-new world orthohantavirus' real-time PCR-assay with Cq = 38, and a 'pan-orthohanta' nested PCR qualitative assay). The 400 nt nested PCR product was sequenced, confirming the presence of ANDV RNA identical to the m/v *Hondius* outbreak strain, excluding infection with a different orthohantavirus species. Results were shared with the WHO and ECDC on 3 June via the Early Warning and Response System.

A subsequent routine weekly monitoring sample collected from HCWx on 26 May tested negative. Instead of hospitalisation, HCWx was monitored at home with twice weekly PCR testing of whole blood, urine and nasopharyngeal swabs, serology and clinical evaluation.

Following extensive expert consultation, this finding was classified as a ‘single low-positive PCR result’, and an ‘inconclusive clinical case'. The quarantine of HCWx ended on 19 June: all follow-up samples during this period remained PCR-negative, serology remained negative and he stayed asymptomatic. The other six nurses tested negative in weekly PCR, and none showed seroconversion at the end of quarantine.

## Assessment of possible transmission routes

On 7 May 2026, HCWx had arrived at the patient’s room approximately 1 h after the patient was transferred to the ICU. This HCWx was not involved in care or transport of the patient. We reconstructed a detailed timeline of events in and around the patient’s room using in-depth interviews and objective timestamp data from telephone calls, messages and electronic hospital records (Figure). To assess the potential for contamination during handling of the patient’s urine around the bedpan disinfector, we simulated this process using fluorescent fluid. An airflow test was conducted in the patient room and the anteroom.

**Figure:**
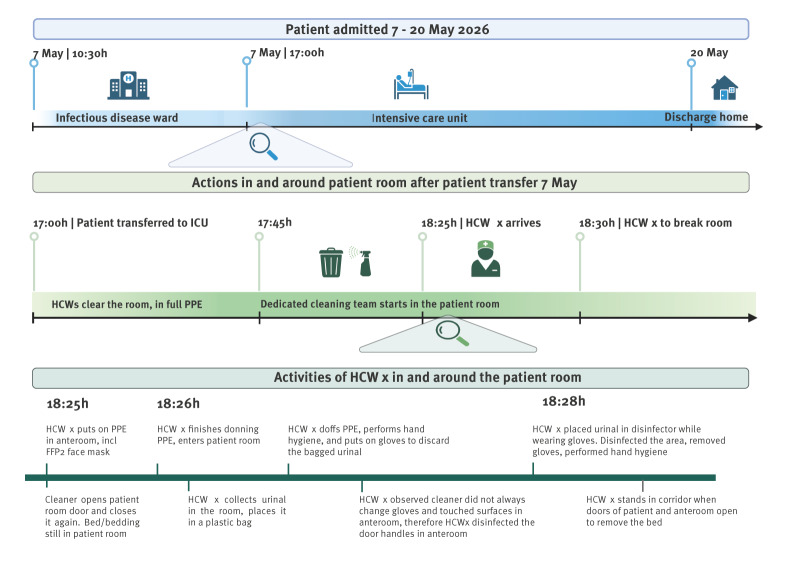
Simplified timeline of the patient’s admission, the actions in and around the patient room and activities of a healthcare worker with positive Andes hantavirus PCR, the Netherlands, 2026

FFP2: filtering face piece (similar to N95); HCW: healthcare worker; ICU: intensive care unit; PPE: personal protective equipment.

This figure visualises the elements of the reconstructed timeline of the chain of events that were considered most relevant for the risk assessment. Clearing the room means that HCWs are supposed to take everything out of the room except furniture, before the dedicated cleaning teams starts their procedure. The urinal should have been removed during that procedure but was still present in the room when the cleaning started. The healthcare worker with the transient low-positive ANDV-PCR test is indicated as HCWx. HCWx was indirectly exposed on 7 May, and the blood sample of HCWx that tested positive for ANDV was taken on 19 May 2026. Created in BioRender.

### Potential exposure to urine

In the patient room on the ID ward, all HCWs, including HCWx, wore PPE according to IPC protocol. On 7 May, a urinal containing the patient’s urine remained in the patient’s ID ward room after the patient was transferred to the ICU. The urinal was placed in a plastic bag and transported through the hallway to the bedpan washer–disinfector in a controlled manner by HCWx who was wearing gloves. This was all done on 7 May and occurred only once after the patient was transferred to the ICU. No splashing occurred, and glove change and hand hygiene was performed correctly. The plastic bag was removed without any splashing and was disposed in a dedicated bin for infectious materials. The fluorescent fluid simulation showed no contamination of the area surrounding the bedpan disinfector that is designed as a closed system to prevent aerosol formation. The bedpan disinfector empties the urine from the urinal automatically after the door is closed. Although the HCW was initially classified as a ‘high risk’ due to possible exposure to the patient’s urine, later investigation deemed this transmission route unlikely.

### Potential airborne transmission

On 7 May 2026, HCWx was present in the anteroom, wearing an FFP2 mask, during brief door openings by the cleaning staff, and earlier on another occasion in the hallway, without any PPE, while doors between the patient room, anteroom and hallway were simultaneously open. Airflow testing confirmed that opening the patient room door results in airflow towards the anteroom, indicating a theoretical risk of airborne contamination. However, this risk was mitigated by at least six air exchanges before HCWx’s presence and the use of an FFP2 respirator in the anteroom and at least 2 m distance in the hallway. Overall, airborne transmission was considered unlikely.

### Potential contact transmission in the anteroom

We identified several potential risk events in the anteroom related to IPC protocol breaches during cleaning activities: the doors were repeatedly left open; cleaning staff moved between the patient room and anteroom without changing gloves or performing hand hygiene; cleaning staff’s personal items (phone, notebook) were handled by the cleaning staff and later tossed outside onto a trolley (it is unclear what happened to these items thereafter); not all patient-related items, including the urinal, were removed before room cleaning; cleaning staff expressed uncertainty about procedures; a plastic bag with potentially contaminated linen was placed on the bed that exited the room. The cleaner wore PPE at all times. These findings suggest that the anteroom was highly likely to have become contaminated, but this was not confirmed by testing the environment for hantavirus. Although HCWx followed PPE procedures, he donned his PPE inside the anteroom, assuming it was clean. Contact transmission in the potentially contaminated anteroom was therefore considered the most likely route of infection. This conclusion was drawn by internal and independent external experts on 19 June, near the end of the quarantine period. 

## Discussion 

We describe an HCW with a transient positive ANDV PCR result that did not manifest in symptomatic disease or seroconversion. Positive ANDV PCR results have been reported in the presymptomatic phase, but RNA positivity without subsequent disease or seroconversion has not been reported in the literature before [6]. Our finding may indicate that asymptomatic ANDV infections with low-level viraemia can occur. One may hypothesise that in this case, the innate immune system had already cleared the low virus load before symptoms could develop and an adaptive immune response occurred [7,8].

Nosocomial transmission of ANDV to HCWs has been described before [9] but in contrast to our case, these were HCWs with direct close contact without using PPE. Our careful timeline reconstruction suggests that full protocol compliance is hard to achieve, while IPC protocol breaches can have major consequences in the context of HCIDs [10]. The incident has been reported by the hospital to the national Labour Inspectorate (Arbeidsinspectie). The hospital is now addressing the detected issues, including a more in-depth analysis of all events and decisions that led to the quarantine of HCWs (ongoing), clarifying responsibilities of involved staff, and optimising the cleaning procedure, including the training of the dedicated cleaning team. A public summary of the analysis has been published in Dutch [11].

Additional safety measures may be considered. While admission to a high-level isolation unit is one option, alternatives in a standard negative-pressure isolation room could include precautionary observation during cleaning or high-risk procedures, additional 'just-in-time' staff training, and interim anteroom cleaning and disinfection. The events in this case emphasises the need for highly trained personnel and secured processes in the management of patients with a (suspected) HCID.

## Conclusion

We describe a HCW with a transient positive ANDV PCR result without subsequent symptoms or seroconversion. Although the exact route could not be determined, contact transmission in the anteroom was considered the most likely explanation. This case highlights that, for the occupational safety of our HCWs and cleaning staff, clear and trained procedures, including for cleaning, are essential in the management of patients with HCID. 

## Data Availability

Not applicable.
